# Regulating immune memory and reversing tumor thermotolerance through a step-by-step starving-photothermal therapy

**DOI:** 10.1186/s12951-021-01011-2

**Published:** 2021-09-30

**Authors:** Lihua Luo, Bing Qin, Mengshi Jiang, Lin Xie, Zhenyu Luo, Xuemeng Guo, Junlei Zhang, Xiang Li, Chunqi Zhu, Yongzhong Du, Ling Peng, Jian You

**Affiliations:** 1 College of Pharmaceutical Sciences, Zhejiang University, 866 Yuhangtang Road, Hangzhou, 310058 Zhejiang People’s Republic of China; 2 Department of Respiratory Disease, Zhejiang Provincial People’s Hospital, Hangzhou, 310003 Zhejiang China

**Keywords:** Immune memory, Thermotolerance, Photothermal therapy, Starving cancer therapy

## Abstract

**Background:**

Photothermal therapy (PTT) is a highly effective treatment for solid tumors and can induce long-term immune memory worked like an in situ vaccine. Nevertheless, PTT inevitably encounters photothermal resistance of tumor cells, which hinders therapeutic effect or even leads to tumor recurrence. Naïve CD8+ T cells are mainly metabolized by oxidative phosphorylation (OXPHOS), followed by aerobic glycolysis after activation. And the differentiate of effector CD8+ T cell (CD8+ T_eff_) into central memory CD8+ T cell (CD8+ TCM) depends on fatty acid oxidation (FAO) to meet their metabolic requirements, which is regulated by adenosine monophosphate activated protein kinase (AMPK). In addition, the tumor microenvironment (TME) is severely immunosuppressive, conferring additional protection against the host immune response mediated by PTT.

**Methods:**

Metformin (Met) down-regulates NADH/NADPH, promotes the FAO of CD8+ T cells by activating AMPK, increases the number of CD8+ TCM, which boosts the long-term immune memory of tumor-bearing mice treated with PTT. Here, a kind of PLGA microspheres co-encapsulated hollow gold nanoshells and Met (HAuNS-Met@MS) was constructed to inhibit the tumor progress. 2-Deoxyglucose (2DG), a glycolysis inhibitor for cancer starving therapy, can cause energy loss of tumor cells, reduce the heat stress response of tumor cell, and reverse its photothermal resistance. Moreover, 2DG prevents N-glycosylation of proteins that cause endoplasmic reticulum stress (ERS), further synergistically enhance PTT-induced tumor immunogenic cell death (ICD), and improve the effect of immunotherapy. So 2DG was also introduced and optimized here to solve the metabolic competition among tumor cells and immune cells in the TME.

**Results:**

We utilized mild PTT effect of HAuNS to propose an in situ vaccine strategy based on the tumor itself. By targeting the metabolism of TME with different administration strategy of 2DG and perdurable action of Met, the thermotolerance of tumor cells was reversed, more CD8+ TCMs were produced and more effective anti-tumor was presented in this study.

**Conclusion:**

The Step-by-Step starving-photothermal therapy could not only reverse the tumor thermotolerance, but also enhance the ICD and produce more CD8+ TCM during the treatment.

**Graphical Abstract:**

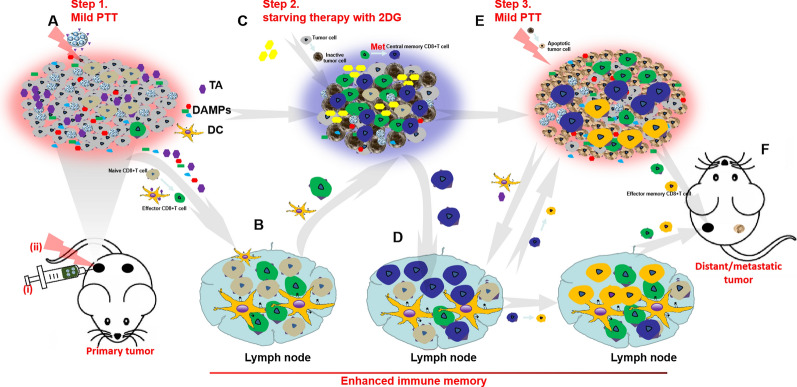

**Supplementary Information:**

The online version contains supplementary material available at 10.1186/s12951-021-01011-2.

## Introduction

Photothermal therapy (PTT), works by converting near-infrared (NIR) light energy into heat, attracted a lot of attention as a non-invasive and localized tumor treatment modality [1–4]. The rapidly raised local temperature is sufficient to smoothly kill tumor cells, without causing obvious wounds and side effects, which makes PTT significantly superior to traditional cancer therapies^5^–^10^. Recently, laser immunotherapy (LIT) in combined with local selective photothermal therapy is increasingly being developed in the treatment of metastatic tumors, since their interaction induces a long-term, systemic anti-tumor immunity via actively stimulating the immune system [11]. LIT combines local selective photothermal interaction and active immunological stimulation to induce a long-term, systemic anti-tumor immunity. The NIR laser and the light-absorbing agent can induce an extremely increase of temperature in the target tissue, which result in a tissue destruction zone covering the target tumor mass selectively.

Oppositely, it actually causes tumor cells swell and break into pieces, releasing tumor antigens, such as tumor-associated antigens (TAA), thermally induced damage associated molecular patterns (DAMPs), and a large number of self-antigens, to activates dendritic cells (DCs). DCs are professional antigen presenting cells (APCs) with the capacity to capture antigen, migrate to the lymph node, and induce T cell-mediated immunity. The LIT-treated tumors in patients serve as potential source of cancer antigens in situ and all the cancer antigens come from the patients’ own tumor cells, such as autologous vaccination. In addition, without in vitro procedure for pre-selection for specific cancer antigens, the whole cell is utilized as a vaccine in LIT, which is conducive to the generation of immune memory and the prevention of tumor recurrence and metastasis [12–16].

To ablate tumors thoroughly, harsh photothermal heating (high temperature over ~ 50 °C) is imperative [17]. Non-localized heat and hyperthermia inevitably result in significant damage to healthy tissues. Therefore, mild hyperthermia (42–47 °C) is usually conducted during the PTT to induce tumor cells death and reduce side effects [18, 19]. Nevertheless, the tumor cells are able to rapidly activate their cytoprotective pathways during PTT treatment, contributing to the resistance to light and heat, which in turn hampers tumor eradication, leading to poor prognosis and high recurrence [20]. Heat shock proteins (HSPs) in particular, is heavily induced to repair thermal damage of proteins and issue in the thermotolerance upon laser irradiation. Thus, thermotolerance has become a major obstacle to PTT of tumors. Recent therapeutic breakthroughs in overcoming the thermotolerance have been achieved by down-regulating the expression of HSPs or using the glycolysis inhibitor [21, 22].

**Scheme 1:**
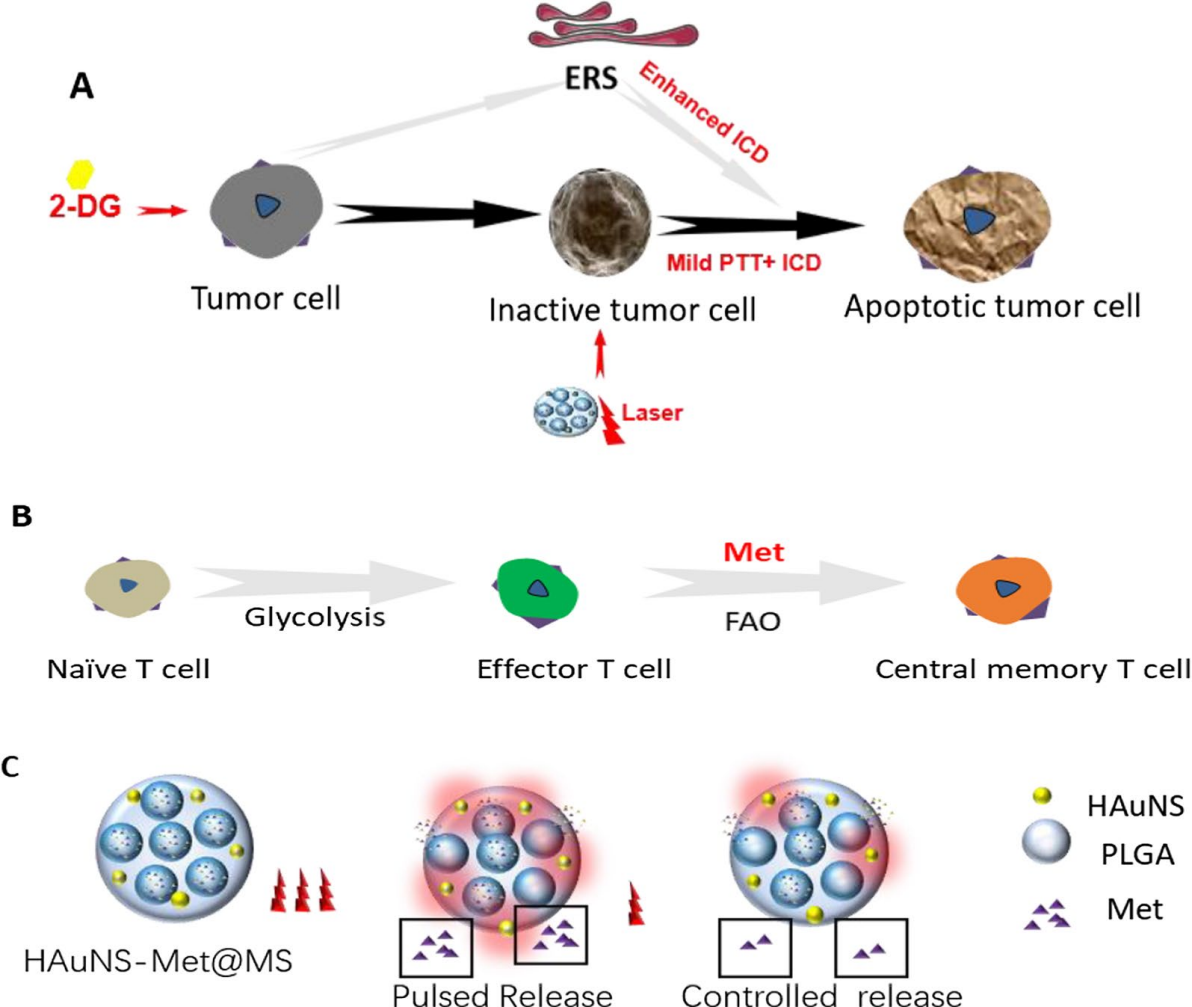
Schematic diagram of therapeutic mechanism. **A** 2DG enhanced the therapeutic effect of PTT through inhibiting the aerobic glycolysis and enhancing the immunogenic cell death (ICD) through inducing the endoplasmic reticulum response (ERS). **B** The subset of T cells experienced different metabolic models. Naïve T cells mainly function in oxidative phosphorylation (OXPHOS), activated effector T cells mainly rely on aerobic glycolysis to obtain energy. And the central memory T cells (TCMs) increase the decomposition of fatty acid oxidation (FAO) to meet the requirement of energy. Metformin (Met) promote the effector T cells differentiation into TCM through increase FAO metabolic pathway. **C** The PLGA microspheres co-loaded HAuNS and Met (HAuNS-Met@MS) maintain a pulsed and controlled long-time release behavior of Met under the different NIR laser

Glycolysis is a major pathway for energy production used for growth and proliferation of tumors and stromal cells in the tumor microenvironment (TME). 2-Deoxyglucose (2DG), a glycolysis inhibitor that can cause cellular energy deficit and death of tumor cells. Therefore, using 2DG to inhibit glycolysis in TME and suppress the synthesis of related stress proteins is promising to reduce the heat stress response and reverse the thermotolerance of tumor cells [23–25]. In addition, 2DG targeting hexokinase can trigger oxidative stress in cancer cells and affect glucose metabolism. By inhibiting protein glycosylation, which is usually considered as a crucial factor in the endoplasmic reticulum stress (ERS) response and an immunogenic cell death (ICD) inducer, metabolic stress can be induced and some signs of ICD are upregulated to enhance antitumor immunity. Moreover, the metabolic competition between tumor cells and T cells is one of the major factors result in tumor immune escape. Inhibition of glycolytic metabolism of tumor cells and their stromal cells by 2DG will benefit the functional activity of immune cells, especially T cells, which are the main force in fighting against tumors [26–28]. Thus, we hypothesized that mild PTT combined with 2DG would not only reverse the thermotolerance of tumor cell, but synergistically enhance the immune response effect induced by PTT (Scheme 1A).

Fate of T cells from naïve to effector or memory phenotype required metabolic remodeling [29]. CD8+ T cells produce energy mainly through oxidative phosphorylation [30], then dominated by aerobic glycolysis when activated. While CD8+ central memory T cells (TCM) mainly achieves their metabolic requirements through fatty acid oxidation (FAO) [31–34], which is controlled by adenosine monophosphate activated protein kinase (AMPK) [35, 36]. Metformin (Met), an oral hypoglycemic drug used in the treatment of type II diabetes, can inhibit mitochondrial complex I, down-regulate NADH/NADPH, activate AMPK, and promote FAO. The use of Met in bacterially infected mice could increase the number of TCM [37–41]. Thus, using Met to differentiate T cells into TCM is expected to enhance the long-term immune memory in PTT-treated mice (Scheme 1B).

For this purpose, we applied PLGA microspheres co-loaded hollow gold nanoshells (HAuNS) and Met (HAuNS-Met@MS) to achieve thermal ablation of primary tumors under the NIR laser. In previous study, we have applied HAuNS, which exhibits plasmon absorption in the NIR region and displays strong photothermal conducting property, to eradicate solid tumors [42,43]. In this work, met encapsulated in the microsphere maintained a pulsed and a long-time release behavior, which could continuously ameliorate the TME and benefit the anti-tumor therapy [16] (Scheme 1C).

**Scheme 2:**
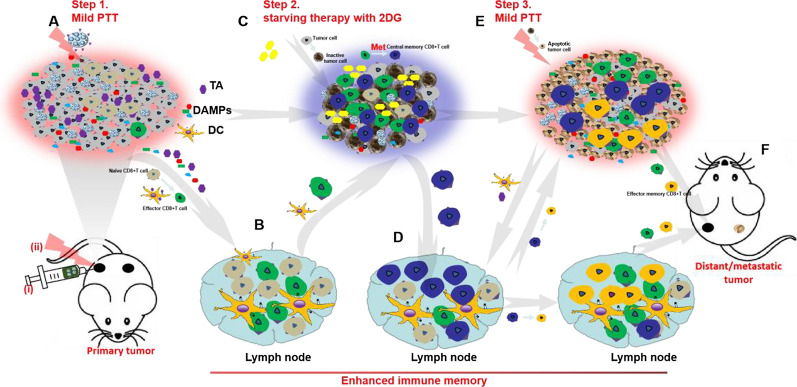
The step-by-step starving-photothermal therapy for distant or metastatic tumor. **A** Step1, Mild PTT was conducted on the primary tumor after injection of HAuNS-Met@MS, which could induce the release of tumor antigen (TA) and DAMPs and mediate the ICD of primary tumor cells. **B** The released TA and DAMPs could promote the activation of DCs and effector T cells. **C** Step2, 2DG was then injected into the primary tumor site after mild PTT to inhibit the glycolytic metabolism of tumor and stromal cells, which could decrease the activity of tumor cells and provide a favorable environment for Central memor CD8+ T cells (CD8 + TCMs) differentiation. **D** CD8 + TCMs was homing to LNs. **E** Step3, Mild PTT on primary tumor was conducted again, based on glycolytic inhibition and ICD enhancement, tumor cells were thoroughly ablated and the distal and metastatic tumor cells were hunted by activated T cells or re-activated CD8+ TCMs. **F** Eradication of distant / metastatic tumor

All in all, a step-by-step treatment strategy through HAuNS-Met@MS in combination with 2DG was designed (Scheme 2) to reverse the thermotolerance of tumor cells, ablate the primary tumor thoroughly, and simultaneously elicit enhanced long-time immune memory to fight against the metastatic tumor. Step one, HAuNS-Met@MS was injected into the primary tumor and supplemented with mild PTT, mediating the release of Met, and inducing the ICD of tumor cells, which recruited DCs and T cells from LNs to the TME (Scheme 2A, B). Step two, 2DG was locally injected to inhibit the glycolysis metabolism of tumor cells, and accompanied by the inactivation of tumor cells, which provided a favorable survival environment for the differentiation of TCMs (Scheme 2C, D). Step three, PTT was conducted to completely eradicate the primary tumor and simultaneously stimulated the release of tumor antigen to activate and attract TCMs, inducing a strong immune response to fight against metastatic tumor cells (Scheme 2E, F).

## Results

### Construction of HAuNS-Met@MS and enhanced cytotoxicity study in combination with 2DG

**Fig. 1:**
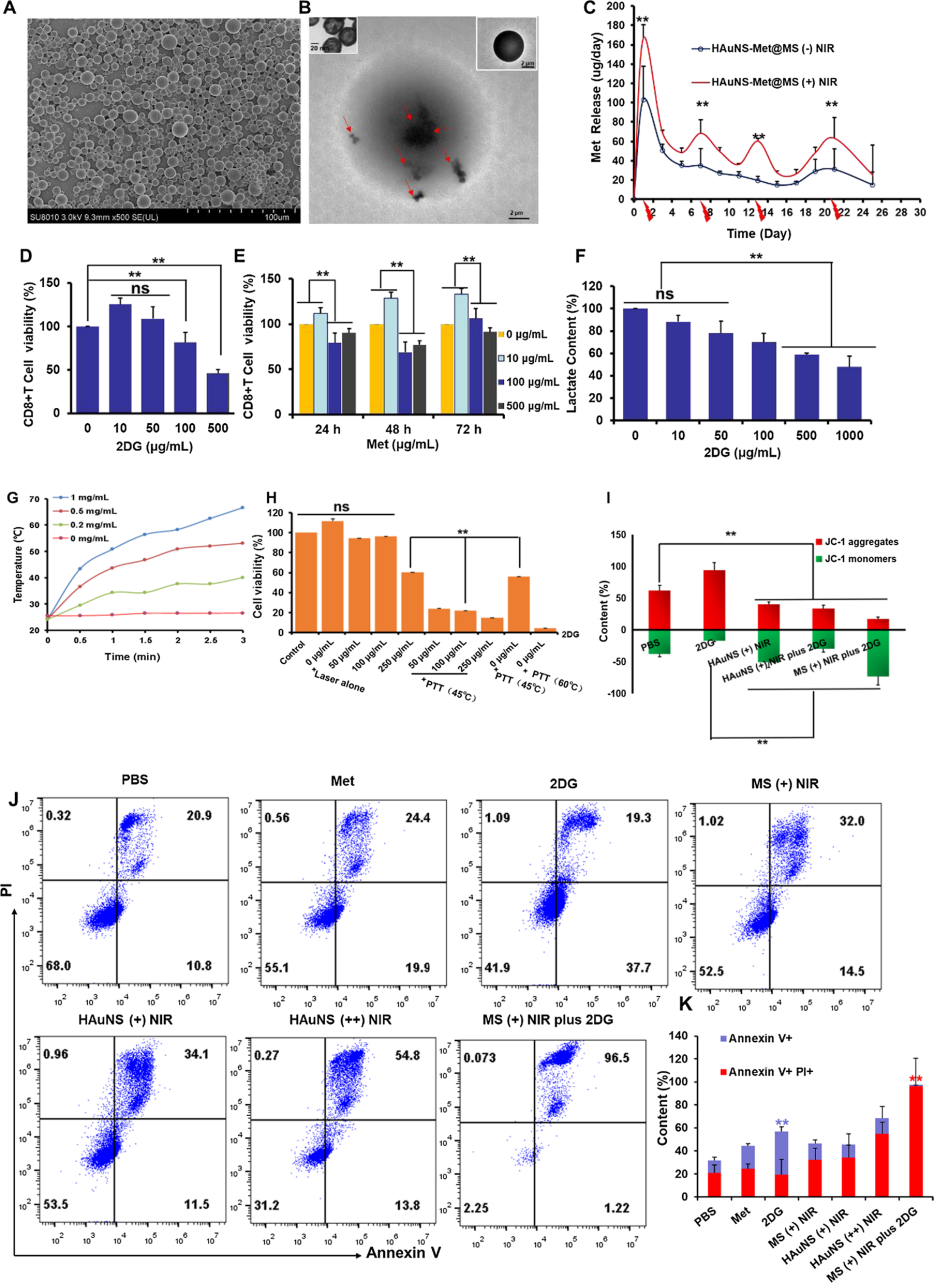
Construction of HAuNS-Met@MS and the enhanced cytotoxicity study of combined treatment with 2DG. **A** SEM image of HAuNS-Met@MS (Scale bar: 100 μm); **B** TEM images of HAuNS-Met@MS (Scale bar: 2 μm), HAuNS (upper left corner, Scale bar: 20 nm), and zoomed picture of HAuNS-Met@MS (top right corner, Scale bar: 2 μm). The red arrows indicate the encapsulated HAuNS. **C** Daily release profile of Met from HAuNS-Met@MS with/without laser irradiation. **D** CD8 + T cell viability after incubation with 2DG at different concentrations. (E) CD8+ T cell viability after incubation with Met at different concentrations for 24, 48 and 72 h. (F) Lactate release ratio of CD8+ T cells at different concentrations of 2DG. (G) Time–temperature curve of HAuNS at various concentrations. (H) Cell viability of B16F10 after various treatments. (I) Mitochondrial membrane potential of B16F10 was detected with JC-1. (J) Representative flow cytometry plots and quantification (K) of apoptosis rates of B16F10 cells. *p < 0.05, **p < 0.01

Previous studies showed that PLGA microspheres loading with HAuNS and small molecule peptide could result in perdurable and controlled release behavior [3, 8, 16, 42, 43]. Here, PLGA microspheres co-loaded with HAuNS and metformin (HAuNS-Met@MS or MS) were constructed. The MS presented a uniform spherical appearance as observed by scan electron microscopy (SEM; Fig. 1A) and HAuNS were encapsulated well in the MS through transmission electron microscopy (TEM; Fig. 1B). The hydrated particle size of MS was 5–10 μm measured by dynamic light scattering. And it was found that HAuNS-Met@MS presented a very low toxicity on 4T1 tumor cells, with over 80% cell viability after 48 h incubation even at 10 mg/mL, indicating that the preparation has good biosafety (Additional file 1: Figure S1A, B). The Met contents in MS were determined to be 60 μg per mg of MS quantified by high performance liquid chromatograph (HPLC). Met displayed prolonged to more than 3 weeks and pulsed release behavior when subjected to NIR laser (Fig. 1C). The daily amount of Met released from MS exceeded 40 μg, which met the requirements of subsequent animal experiments. The proliferation of CD8+ T cells reached 54 million within 6 days after anti-CD3 and anti-CD28 activation (Additional file 1: Figure S3). When incubated with 2DG at low concentration (10 μg, 50 μg), CD8+ T cells displayed a slight increased cell viability. While at 500 μg/mL, the viability of CD8+ T cells were severely reduced. The extra- and intra- cellular lactate of cancer cell (B16F10), as a characteristic metabolite of glycolysis, was significantly decreased in 24 h when incubated with 2DG. At high 2DG concentration (1000 μg/mL), the content of lactate was reduced by 50%, demonstrating that 2DG was efficiently suppressed the hyperglycolytic state of cancer cells (Fig. 1F). We then investigated the cytotoxicity of 2DG and Met on tumor cells [44]. The half maximal inhibitory concentration (IC50) of 2DG and Met on B16F10 cells was 1372 μg/mL and 5548 μg/mL, respectively (Additional file 1: Figure S4). The temperature of HAuNS after irradiation increased with rising concentration (from room temperature to nearly 40 ~ 70 °C in 3 min with NIR irradiation) (Fig. 1G). According to the heating curves, we conducted the PTT effect on B16F10 in combination with 2DG. As shown in Fig. 1H, cells administrated with laser alone meant that they were subjected to NIR laser without incubation of HAuNS, and the temperature of cell culture medium maintained at 37 °C. Mild PTT or strong PTT means that cells were incubated with 1 mg/mL HAuNS and subjected to NIR laser, and the temperature of cell culture medium maintained at 45 or 60 °C, respectively. The results showed that strong PTT killed more than 80% of tumor cells. While mild PTT produced up to 50% cytotoxicity, which was further enhanced with the addition of 2DG, suggesting that the inhibition of the energy metabolism (mainly glycolysis) was capable of significantly promoting the sensitivity of tumor cells to PTT. We next found that the mitochondrial potential of tumor cells (B16F10) was increased by 2DG, which subsequently plummeted by PTT (Fig. 1I and Additional file 1: Figure S5). The same phenomenon was observed in EG7-OVA cells (Additional file 1: Figure S6A–C). Cell death pathways were further investigated in detail by flow cytometry, both B16F10 and EG7-OVA presented distinct late apoptosis in MS ( +) plus 2DG group (96.5, 84%), while MS ( +) NIR, HAuNS ( +) NIR and 2DG groups showed little late apoptosis, confirming that the mitochondrial pro-apoptotic pathway was responsible for the significantly decreased viability of cancer cells after MS ( +) plus 2DG treatment (Fig. 1K-I and Additional file 1: Figure S7A, B).

### 2DG and Met promoted differentiation of CD8+ T cells toward to a memory phenotype

**Fig. 2:**
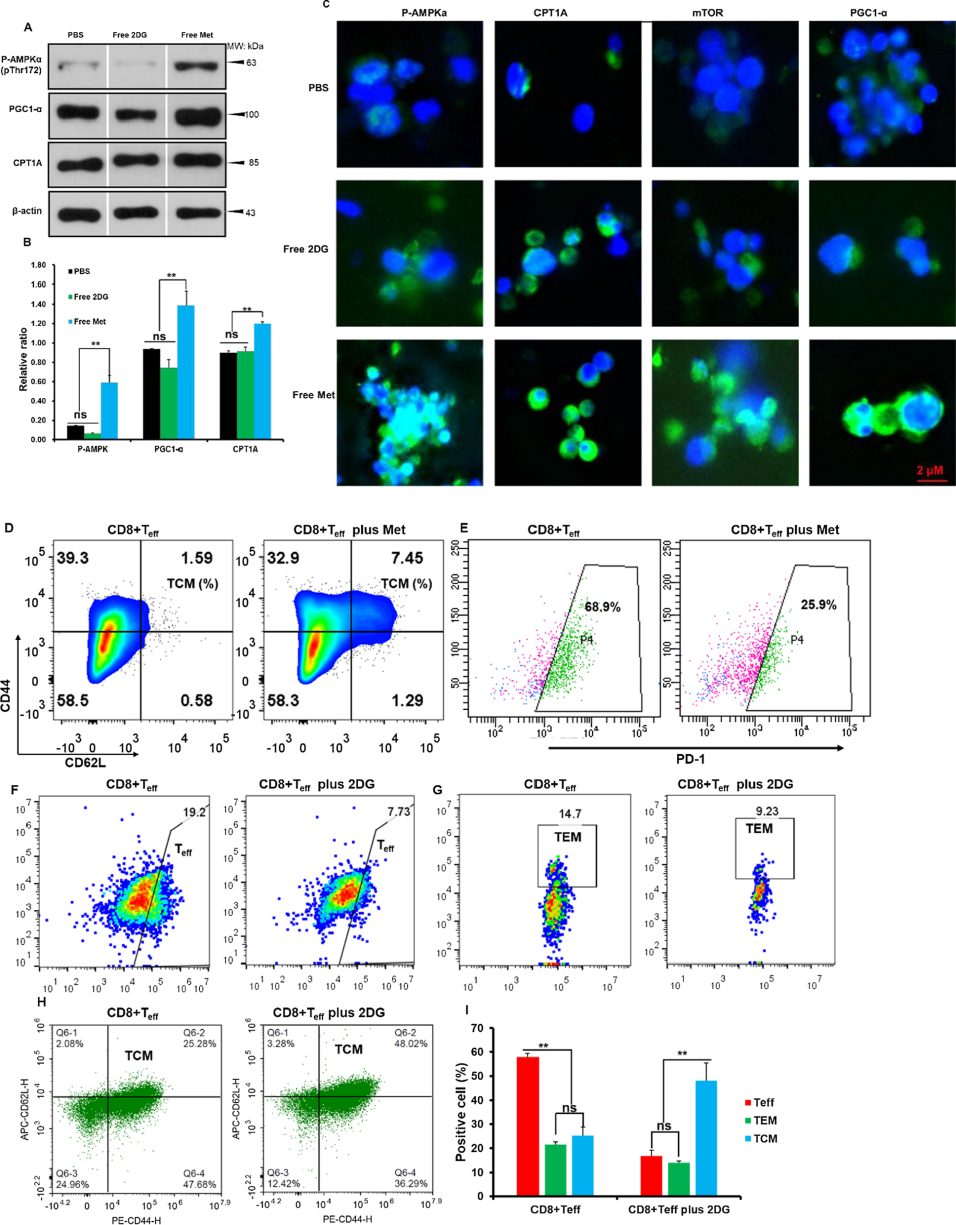
2DG and Met promoted the differentiation of CD8+ TCMs. Western blotting (**A**) and its quantitation (**B**) of P-AMPKα, PGC1-α, CPT1A. **C** Immunofluorescence images of AMPKα, PGC1-α, CPT1A (Scale bar, 2 μm). Flow cytometry analysis of CD8+ T_eff_ central memory phenotypes (**D**) and its PD-1 expression (**E**) on CD8+ T_eff_ cells with Met. Flow cytometry analysis of CD8+ T_eff_ cells (**F**), CD8+ TCM (**G**), CD8+ TEM (**H**) and its quantitation (**I**) with 2DG. *p < 0.05, **p < 0.01

When T cells differentiate to their memory phenotype, the main metabolic pathway changes from aerobic glycolysis to FAO. Targeting cellular metabolism is capable of interfering with the metabolic patterns, thereby promoting the differentiation of CD8+ T cells into memory phenotype, which can be utilized to design as an *in-situ* vaccine. To verify the impact of our strategy on cellular metabolism, the expression of FAO-associated proteins was detected. P-AMPK, PGC1-α [45, 46] and CPT1A was remarkably upregulated with the incubation of free Met (10 μg/mL), significantly higher than that in free 2DG and PBS groups detected by WB and immunofluorescence (Fig. 2A–C). With the addition of 10 μg/mL Met, the CD44 + CD62L + population of effector CD8+ T cells (CD8+ T_eff_) increased by 7.45% and the PD-1 expression was down-regulated, suggesting that Met might have potential anti-tumor activity (Fig. 2D, E). By inhibiting the aerobic glycolysis of T cells with 2DG (10 μg/mL), the expansion of CD8+ T_eff_ and effector memory T cells (TEM) was extremely reduced (Fig. 2F, G). Simultaneously, more CD8+ T_eff_ toward a central memory T cell phenotype (Fig. 2H, I). In conclusion, the combination of 2DG and Met showed a synergistic effect in vitro, interfering with the metabolism, transforming CD8+ T cells into memory phenotypes, and providing a solid foundation for subsequent in vivo experiments.

### Enhancement of ICD and reversion of thermaltolerance in vivo

**Fig. 3:**
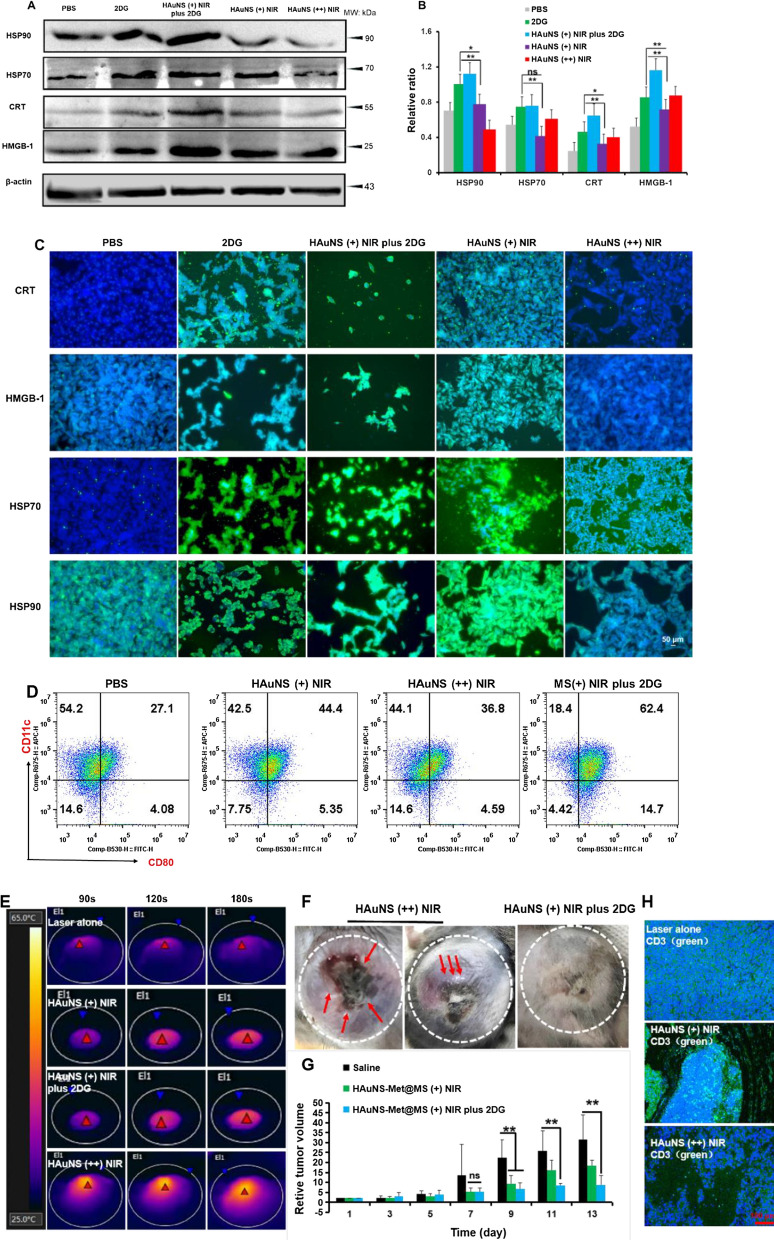
Enhancement of tumor cell ICD and reversion of thermotolerance in vivo. Western blotting (**A**) and its quantitation (**B**) of DAMPs proteins (HSP90, HSP70, CRT and HMGB-1). **C** Immunofluorescence images of the above proteins (Scale bar, 50 μm). **D** Flow cytometry of CD11c + CD80 + DCs. **E** Near infrared thermal images of B16F10 tumor bearing mice. **F** Tumor pictures of mice after mild or strong PTT treatment. **G** Average relative tumor volume of mice. **H** Representative immunofluorescence images of T cell infiltration in tumor tissue. (Blue, nucleus; Green, CD3; Scale bar, 100 μm). *p < 0.05, **p < 0.01

2DG inhibits the protein N-glycosylation pathway in the ER and thus induces the ERS, which further promote ICD with PTT. Less DAMPs in B16 tumor cells were displayed after administration with 2DG or PTT individually, while enhanced protein expression was presented when co-treated with PTT and 2DG, indicating that 2DG played a synergistic effect on inducing ICD of tumor cells when they subjected to PTT (Fig. 3A–C). The upregulation of DAMPs related proteins triggers the “eat me” signal by binding to the surface maker CD91 on DCs, and therefore stimulates the DC maturation and antigen presentation for subsequent immune responses [47]. Thus, DC maturation after different treatments was assessed by analyzing CD80+ and major histocompatibility complex I (MHC I +) using flow cytometry. The CD80 + DCs and MHCI + DCs jumped steeply to 47.5% and 30.5% in the co-treated group compared to 25.7% and 24.25 in the saline group, respectively (Fig. 3D and Additional file 1: Figure S8). Outstanding and controllable NIR photothermal conversion efficiency of HAuNS was displayed in Fig. 3E. To ablate tumors thoroughly, harsh photothermal heating (high temperature over ∼50 °C) was required to induce complete cell necrosis. However, due to inevitable heat diffusion, high temperature treatments might threaten the healthy tissues and cells nearby. Severe skin damage was observed in tumor and H&E staining of PTT (s) groups (Fig. 3F and Additional file 1: Figure S9). A malignant tumor growth around the laser lesion after the PTT treatment was also observed, indicating there was a strong resistance to PTT, which was reversed by co-treated with 2DG. After one week’s treatment, tumor was dramatically inhibited by mild PTT in combination with 2DG (Fig. 3G). The “hyperthermia range” (42–47 °C) resulted in the degeneration of DAMPs related proteins and could further induce immune response. The increased T cell infiltration into the TME preliminarily validate this idea (Fig. 3H). To sum up, 2DG could enhance the ICD of PTT and reverse thermaltolerance in vivo.

### In vivo training of antigen specific memory CD8+ T cells with* in-situ* vaccine

**Fig. 4:**
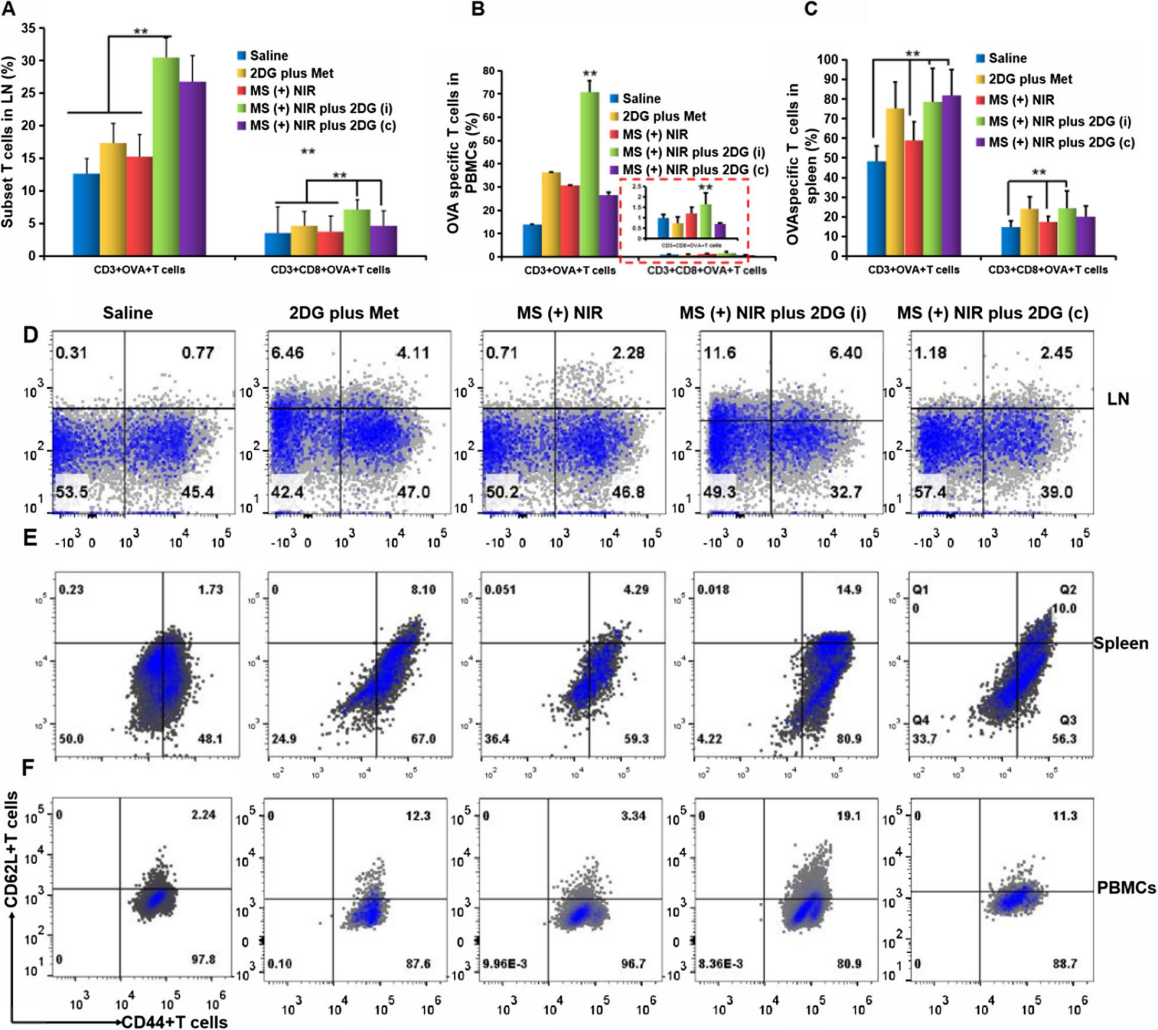
Enhanced antigen specific memory CD8+ T cells in vivo. Quantitation of OVA specific CD3+ or CD8+ T cells in LNs (A), PBMCs (B), and spleen (C). The picture in the red dotted box is an enlarged one of CD3 + CD8+ OVA + T cells of EG7-OVA mice based on flow cytometry analysis. (D-F) Flow cytometry analysis of CD62L+ CD44+ T cells (TCMs) in LNs (D), PBMCs (E), and spleen (F) gated on OVA+ CD3+ T cells. (G-H) CD3 + CD8+ T cells (G) and OVA specific CD8+ T cells (H) infiltrated in tumor. *p < 0.05, **p < 0.01

2DG inhibits tumor cells proliferation by inhibiting glycolytic metabolism, which might also interfere with the function of T_eff_ in the TME (Fig. 2E, F). The interference of 2DG on T_eff_ was supposed to exclude, since it produced energy though glycolytic metabolism in the TME and increased the number of memory T cells. Therefore, we applied different dosing regimens of 2DG including intermittent administration (i, injection of 2DG every 3 days) and continuous administration (c, injection of 2DG every day) to investigate tumor cell glycolysis in the TME and enhancement of memory T cells. HAuNS-Met@MS with slow-release effect was injected intratumorally once and administrated with multiple NIR irradiation (Additional file 1: Figure S10). EG7-OVA bearing mice were administered for 3 weeks. An increase of matured DC (CD11c + CD86 + DC) and OVA specific matured DC (CD86 + MHC-OVA + DC) in lymph nodes (LNs) were observed (Additional file 1: Figure S11A, B), especially in MS ( +) NIR plus 2DG (i) group. T cells within the gated subsets were shown in Additional file 1: Figure S12A–C, and it was found that MS ( +) NIR in combination of 2DG (i) played a positive role in the proliferation of CD8+ T cells. Comparing the mice in MS ( +) NIR plus 2DG (i) group with those in saline group, there were approximately 10% more CD3 + T cells and 20% more CD8+ T cells in LNs (Additional file 1: Figure S12B, C and Fig. 4A). Furthermore, the OVA specific T cells (OVA + CD3 + T, OVA + CD8+ T) were significantly increased in LNs (Fig. 4A). It was also found that there was a notably higher percentage of OVA specific CD3 + T cells and OVA specific CD3 + CD8+  T cells in PBMCs and spleen (Additional file 1: FigureS13 and Fig. 4B, C). CD8+ TCMs are important indicator of *in-situ* vaccination. Thus, TCMs in LNs, spleen and PBMCs tissues of mice after immunization were analyzed by flow cytometry. The specific OVA CD3+ and CD8+ TCMs in LNs were 6.4 and 15% after treated with MS ( +) NIR plus 2DG (i), respectively, which were significantly higher than those in other groups (Fig. 4D). The proportion of specific OVA CD3 + and CD8+ TCMs in spleen were both significantly higher in the MS ( +) NIR plus 2DG (i) group than that in other controls (Fig. 4E). The same tendency was observed in PBMCs and spleen (Fig. 4F). The treatment modality we adopted could successfully induce the infiltrating T cells, and the amounts of OVA specific CD8+ T cells elevated threefold (Additional file 1: Figure S14 and Fig. 4G, H).

### Anti-metastatic efficacy of MS ( +) NIR plus 2DG in EG7-OVA tumors

**Fig. 5:**
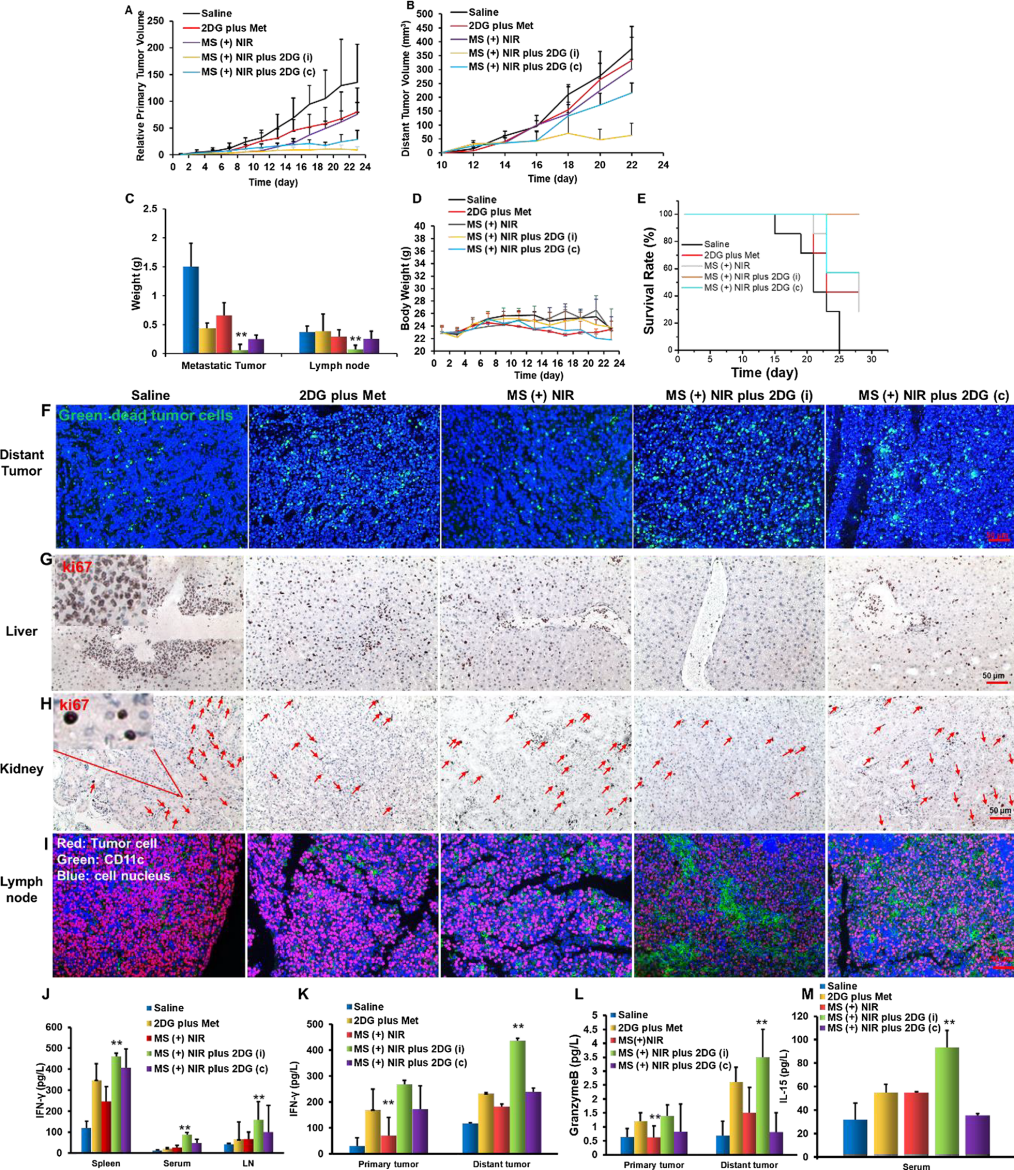
Antitumor efficacy with the step-by-step starving-photothermal therapy in EG7-OVA tumor model. Primary tumor (**A**) and distant tumor (**B**) volume. **C** Weight of metastatic tumors and lymph nodes. Body weight changes (**D**) and survival rate (**E**) of mice. **F** Immunofluorescence of TUNEL in distant tumor. **G**, **H** Immunohistochemistry of Ki67 in liver and kidney. The red arrows represent proliferating tumor cells. **I** Immunofluorescence of lymph node. **J**–**M** Various cytokine expressed in different tissues. *p < 0.05, **p < 0.01

The antitumor effect of MS (+) NIR plus 2DG working as an *in-situ* tumor vaccine was investigated. We established EG7-OVA tumor model on C57 mice (Additional file 1: Figure S15) and treated them according to the scheme in Additional file 1: Figure S10. Both the primary and distant tumor presented a rapid growth in the control groups (Saline, 2DG plus Met and MS ( +) NIR), while a remarkable inhibition was observed in MS ( +) NIR plus 2DG group in the primary tumor (Fig. 5A). A stronger suppression of distant tumors was displayed in MS ( +) NIR plus 2DG (i) group compared with those in MS ( +) NIR plus 2DG (c) group, suggesting that intermittent administrations of 2DG could contribute to a better activation of immunity (Fig. 5B). 21 days later, distinct metastases nodules with the heaviest weight were found in mice of control group. Interestingly, different modes of administration of 2DG brought different degree of inhibition of tumor metastasis. Compared with continuous administration, intermittent administration with 2DG could activate the immunity of mice more efficiently, thereby inhibiting tumor growth and metastasis (Fig. 5C). The body weight of the mice showed no obvious changes during treatment (Fig. 5D), indicating that the therapy had no significant side effects. The activation of immunity prolonged the survival of mice (Fig. 5E). TUNEL assay of distal tumors sections showed that the tumor cells in the treatment groups exhibited the strongest apoptosis (Fig. 5F). It was observed that mice in the control group developed remarkable visceral metastasis, which was dramatically inhibited in the treatment group (Fig. 5G, H). Immunofluorescence analysis of the LNs also revealed that most of the LNs in the control group were occupied by tumor cells, while the treatment group were mostly APC cells (Fig. 5I). The ability of MS ( +) NIR plus 2DG (i) to awake immune response could be further verified by cytokines of spleen, serum and LNs. As shown in Fig. 5J–M, a higher level of IFN-γ in spleen, serum and LNs, and more expression of granzyme B in tumors was observed in treatment groups. As a critical cytokine for the proliferation of T cells, IL-15 level in serum was remarkably increased in all intervention groups. All the results supported the conclusion that our strategy could effectively inhibit tumor growth and metastasis by activating the immune system of mice.

### Anti-tumor efficacy and immune response in B16F10 tumor model

**Fig. 6:**
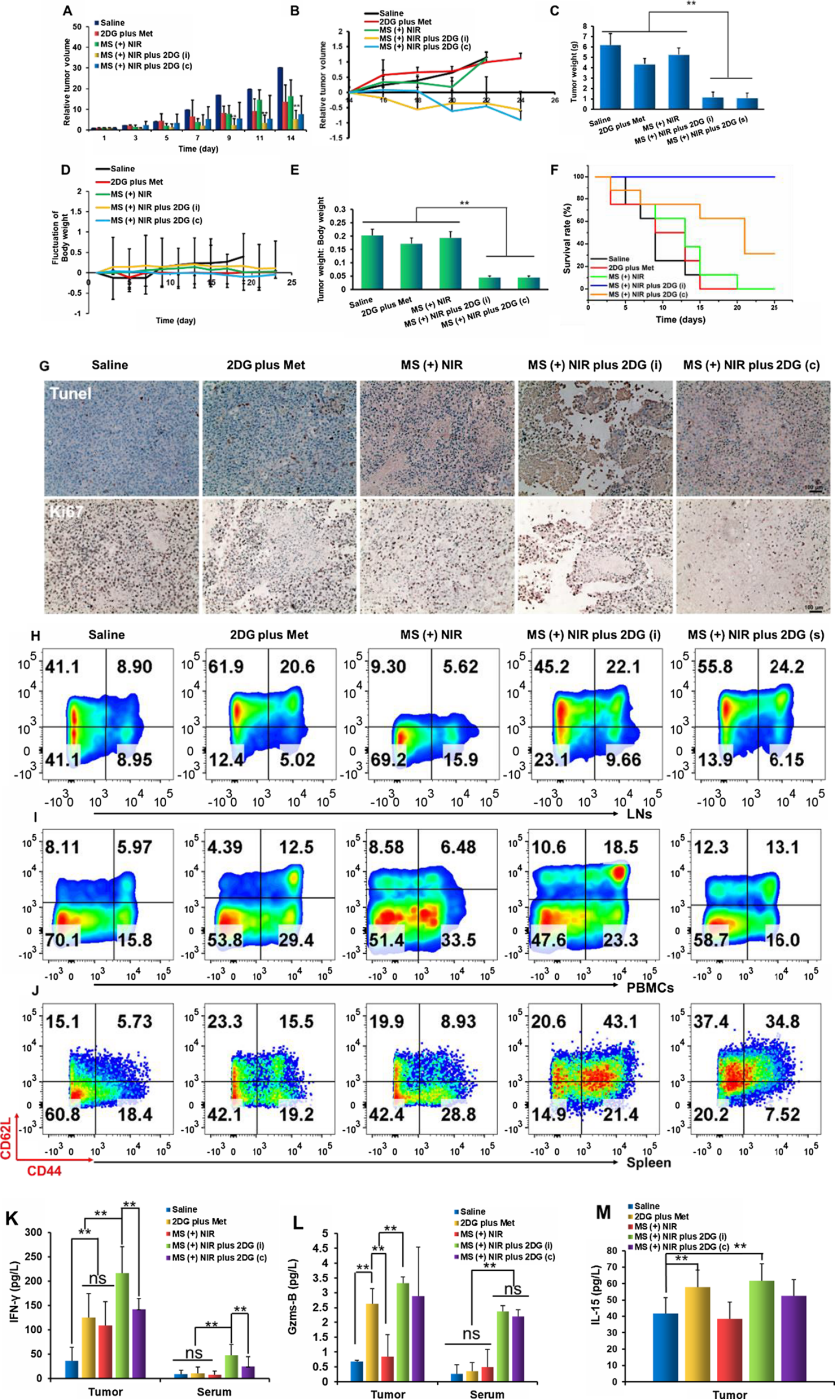
Antitumor efficacy and immune response in B16F10 tumor model. Relative tumor volumes of mice in each group within 14 days (**A**) and tumor volumes of the remaining mice (**B**). **C** Tumor weight of mice in different groups. **D** Body weight changes of mice. **E** The ratio of tumor weight to body weight after different treatments. **F** Survival curves of the tumor-bearing mice after 4 weeks treatments. **G** Ki67 and TUNEL of tumor tissues. Scale bars, 100 μm. (H–K) The proportion of central memory CD8+ T cells in LNs (**H**), PBMCs (**I**) and spleen (**J**). **K**–**M** The proportion of IFN-γ (**L**) and granzyme B (**M**) in tumor tissues and serum

After investigating the antigen specific tumor model (EG7-OVA), B16F10 tumor model was then established to study the broad-spectrum antitumor effects with our step-by-step co-therapy strategy. Tumor model and the administration method were conducted as Additional file 1: Figures S10 and S16. The treatment with MS ( +) NIR plus 2DG showed the best ability to suppress tumor growth, being dramatically higher (P < 0.01) than that of the control group (Fig. 6A). Half of the mice were sacrificed after 2 weeks treatment, and the related immune cells were later investigated. Tumor volumes of the remaining mice were monitored continually and it was found that the MS ( +) NIR plus 2DG remarkably suppressed tumor growth (Fig. 6B). Tumor weights in the MS ( +) NIR plus 2DG group were significantly slighter than those in other groups (Fig. 6C). Body weight of mice in the saline group was obviously increased, while no notable change was found in the other groups (Fig. 6D). Figure 6E displayed the ratio of tumor weight to body weight, suggesting that the weight gain of mice in the saline group mainly related to the enlargement of tumor. Although both MS ( +) NIR plus 2DG (i) and MS ( +) NIR plus 2DG (c) similarly suppressed tumor growth, the mice in MS ( +) NIR plus 2DG (i) group conspicuously presented better survival rates (Fig. 6 F, 100% of the mice survived within 4 weeks). H&E, Ki67 and TUNEL assays showed that the tumor cells in the MS ( +) NIR plus 2DG group exhibited the lowest proliferative activity (Fig. 6G and Additional file 1: Figure S15). The activated immune systems contributed to the better survival rates of mice, in which an obvious increase of CD8+ T cells in spleen, LNs and blood in treatment groups was observed (Additional file 1: Figure S18A–C). Besides, a higher proportion of CD8+ TCMs was observed in our treatment group (Fig. 6H-J). The infiltration of T cells in tumors also revealed that there were denser CD8+ T cell and less regulatory T cells (Treg) in MS (+) NIR plus 2DG (i) group. Meanwhile, functional IFN-γ + CD8+ T cells in treatment group was significantly higher than other groups (Additional file 1: Figure S19). Immune factor including IFN-γ, granzyme B and IL-15 in both tumor tissues and (or) serum were also elevated (Fig. 6 K–M). All of our results indicate that the immune system of the mice was remarkably enhanced after treatment with MS ( +) NIR plus 2DG (i).

## Disscussion and conclusions

PTT can improve the immunogenicity and endogenous adjuvant effect of tumor cells through the photothermal effect, and transform tumor into vaccine factory [48, 49], effectively eradicating the primary tumor and inhibiting tumor metastasis. PTT was one of the best ways to induce in situ vaccines currently [50]. Mild PTT was generally used to treat cancers, which could promote the infiltration of T cells in the tumor tissues (Fig. 3H) [51]. Unfortunately, mild PTT could not completely ablate the tumor tissue and would easily lead to thermotolerance of tumor cells, which might make tumor cells more likely to transfer to the other organs, due to vasodilation caused by PTT^52^,^53^. Therefore, it is key point to choose appropriate drugs to solve the thermotolerance of tumor cells induced by mild PTT. At present, clinical researches often use heat stress protein inhibitors to overcome this problem [54]. In this study, the glycolysis inhibitor 2DG was used to cut off the energy supply of tumor cells and reduced the expression of heat stress proteins, thereby reversing the thermotolerance of tumor cells (Fig. 1H and Fig. 3F, G). In addition, 2DG, known as an ERS inducer, could also amplify the ICD effect induced by PTT (Fig. 3A–D).

Besides, the severe immunosuppressive microenvironment of tumor cells results in the depletion of most effector CD8+ T cells during PTT treatment, and only a small part of CD8+ T cells can differentiate into memory CD8+ T cells. Once activated, naive T cells differentiate into effector T cells and lead to a rapidly promotion of aerobic glycolysis. While the metabolism of central memory T cells preferentially utilize FAO, but does not exclusively depend on aerobic glycolysis. AMPK is a key metabolic regulator in T cells as a metabolic stress sensor, which was reported to be closely related to the formation of central memory cells. Met (an AMPK activator) was utilized to promote the differentiation of CD8+ TCM, which could also relieve the hypoxia of TME [55, 56], down-regulate PD-L1 protein on tumor cells[57], and decrease the expression of PD-1 on T cells (Fig. 2E). That is, Met exerted a positive effect on regulating the immune microenvironment and improving the infiltration of T cells in tumor tissues. Here, a sustained drug release vector prepared with PLGA was designed to reduce the frequency of administration. Our data indicated that the peptide can maintain a long and sustained release up to 3 weeks. Furthermore, HAuNS-Met@MS vector constructed in this study could control the release of Met through the photothermal effect, and had a long-term improvement effect on the TME through perdurable release of Met (Fig. 1C).

In addition, because both effector and effector memory T cells depend on glycolysis for energy metabolism like tumor cells, 2DG might also affect the activity of effector T cells (Fig. 2F–I), so different 2DG dosing interval was explored to ensure that 2DG mainly acted on tumor cells. From the results of in vivo experiments (Fig. 4), it was found that intermittent administration of 2DG in combination with HAuNS-Met@MS could contribute the highest proportion of OVA-specific CD8+ T cells as well as CD8+ TCMs in the tumor tissues of mice, and even significantly inhibited the primary tumor and metastasis. (Figs. 5 and 6). To sum up, in this study, we used mild PTT effect of HAuNS to propose an in situ vaccine strategy based on the tumor itself. By targeting the metabolism of TME with different administration strategy of 2DG and perdurable action of Met, the thermotolerance of tumor cells was reversed, more CD8+ TCMs were produced and more effective anti-tumor was presented in this study.

## Experimental section

### Materials

All the chemical reagents were purchased from Aladdin Biochemical Technology Co.,Ltd. (Shanghai, China) unless otherwise specified. Poly (D, L-lactic-co-glycolide) (PLGA, 50:50 (w:w), MW: 50 k Da) was from Dai Gang biological company (Ji Nan, China). Metformin hydrochloride (Met) and 2-Deoxy-D-glucose (2DG) were obtained from Meilun Biotechnology Co., Ltd (Dalian, China). All reagents used for western blotting were acquired from Proteintech Group, Inc (Rosemont, USA). Carboxyfluorescein succinimidyl ester (CFSE), FITC-anti-CD3 antibody and other antibodies against cell surface markers for flow cytometry (fluorescent-activated cell sorting) assay were purchased from eBioscience (California, USA). T-Select MHC Tetramer was purchased from Beijing B&M biotech co., LTD (Beijing, China), Fetal bovine serum (FBS), RPMI 1640 medium, DMEM medium, and trypsin–EDTA were purchased from Ginuo biotech co., LTD (Hangzhou, China).

### Cells and animals

Murine colorectal tumor CT26 cells, melanoma B16F10 cell and EG7-OVA cell lines were originally obtained from the Institute of Biochemistry and Cell Biology (Shanghai, China) and cultured in 25 cm^2^ flasks at 37 °C in a humidified atmosphere containing 5% CO_2_. Peripheral blood mononuclear cells (PBMCs) or splenic lymphocytes were isolated using lymphocyte density gradient centrifugation with Ficoll-paque PREMIUM. T cells were enriched with magnetic microbeads (Miltenyi Biotec). Bone marrow derived dendritic cells (BMDCs) were generated from the bone marrow of 8-week-old C57BL/6 mice according to an established method [16, 43]. Naïve T cells were activated with αCD3 (1.0 μg/ml), αCD28 (0.5 μg/ml), and 100U/ml of IL-2 for 3 or 4 days and then assayed as indicated (Shanghai, China) and cultured in 25 cm^2^ flasks at 37 °C in a humidified atmosphere containing 5% CO_2_.

C57BL6 mice (18–20 g, 6–8 week, male) were housed in appropriate animal facilities at Zhejiang University. In vivo experiments were performed in compliance with the requirements of the Zhejiang University Animal Study Committee for the care and use of laboratory animals in research. In the relevant animal experiment license mice were regarded as death when tumors exceeding 35,00 mm^3^.

### Preparation and characterization of HAuNS-Met@MS

HAuNS were synthesized and hydrophobically modified with octadecyl-3-mercaptopionate as previously reported [3, 8, 16, 42, 43]. 200 mg PLGA was dissolved in 4 mL dichloromethane containing 0.2 mg hydrophobic modified HAuNS, which was used as oil phase. 10 mg Met was taken to dissolve in 200 μL cold PBS as inner water phase. HAuNS-Met@MS were prepared through W/O/W double emulsion method and purified by centrifugation. After that, the microspheres were freeze-dried into microspheres. Drug encapsulation efficiency was detected by high performance liquid chromatography (HPLC). The HPLC analysis was performed using Agilent system. A C18 5 μm, 4.6 × 250 mm column (Agilent, USA) was used to separate Met. The mobile phase consisted of acetonitrile (Merck, Germany) and phosphate-buffered solution (Sigma Co, USA) (pH 7.0) (70:30 v/v). The standard curve of Met was displayed as Additional file 1: Figure S2. The morphology of HAuNS-Met@MS was characterized by scanning electron microscope (SEM) (JEOL 7600F, Hitachi, Japan) and TEM (JEOLJEM-1230, Japan).

### Drug release profile

For Met release study, 3 different batches of HAuNS-Met@MS (100 mg) was suspended in 10 mL of PBS (pH 6.8) respectively which was then divided into two parts and stirred under 37 °C with shaking at 500 rpm. One of the MS was received NIR laser at the predetermined time with an 808 nm NIR laser at an output power of 1.5 W/cm^2^ for 3 min (wavelength of 808 nm, Diomed 15 plus, Cambridge, UK). The other MS was set as control. A total of 0.1 mL of the solution was collected at the predetermined time, followed by centrifugation (12 000 rpm for 10 min). The Met concentration in the supernatant was analyzed using high performance liquid chromatography (Agilent 1260, Gremany).

### In vitro cellular experiments

First, the half maximal inhibitory concentration (IC50) of 2DG and Met was calculated on B16F10. And the cytotoxicity of 2DG and Met with various concentrations on CD8+ T cells and B16F10 were evaluated by a Cell Proliferation Assay Kit. Lactate content of tumor cells (B16F10) after incubation with 2DG was tested with L-Lactate Assay Kit (EnzyChrom™). A NIR-laser-mediated rise in temperature and PTT combined with 2DG induced cytotoxicity of tumor cells were measured. Briefly, the solutions containing free HAuNS were used to evaluate the NIR laser-mediated rise in temperature during 3 min with an output power of 0.5 W/cm^2^. The temperatures of the solutions were detected with an electronic thermometer. According to heating curve, the B16F10 cells were treated with mild PTT and strong PTT, with which the temperature was set at 45 and 60 °C respectively. And in combination with 2DG at different concentration, the cytotoxicity of B16F10 was measured with MTT assay.

To evaluate the variation of mitochondrial membrane potentials after different treatments: PBS, free 2DG (100 μg/mL), HAuNS( +) NIR (Mild PTT conducted with free 10 μg/mL HAuNS, ~ 45 °C), HAuNS( +) NIR plus 2DG, and MS ( +) NIR plus 2DG (Mild PTT conducted with MS containing free 10 μg/mL HAuNS, ~ 45 °C). B16F10 and EG7-OVA cells were washed with PBS for 3 times and incubated with JC-1 (Beyotime, C2006) for 20 min, followed by washing with buffer solution twice. The fluorescence of tumor cells at 488 nm (for JC-1 monomer) and 525 nm (for J-aggregates) were monitored using a fluorescence microscope (Nikon, Japan). And the absorbance of fluorescence was further quantified with flow cytometry. B16F10 and EG7-OVA cells were incubated in 6-well plates for 24 h. After the indicated treatments, cells were trypsinized, washed and resuspended in 0.5 mL of binding solution, followed by the incubation with the Annexin V-FITC/PI Apoptosis Detection Kit (Beyotime, C1062S) in the dark for 15 min. Then cells were rinsed with PBS twice and analyzed immediately using a flow cytometer (BD FACSCalibur, USA).

CD8+ T cells were isolated from splenic lymphocytes of healthy mice with magnetic microbeads and activated with anti-CD3 and anti-CD28 for 3 days, after that, the activated CD8+ T were treated with 2DG and Met for a week. Then cells were collected and labeled with flow cytometric antibodies such as PD-1, anti-CD44 and anti-CD62L to analysis the expression of related protein.

### In vivo study 2DG reversed the thermotolerance of B16F10

To investigate the different PTT in tumor under NIR laser irradiation, mice bearing B16F10 tumors were intratumorally injected with saline (100 μL), free HAuNS-Met@MS (containing 0.2 mg HAuNS) or HAuNS-Met@MS plus 2DG (4 mg). The temperature in the tumors under NIR laser irradiation (2 W/cm^2^) was controlled and recorded using a forward-looking infrared (FLIR) thermal camera (Thermal CAM, USA). After irradiation for 3 days at 45 °C, tumor volume was recorded.

### Training of OVA specific memory CD8+ T cells in vivo with* in-situ* vaccine

Mice were injected subcutaneously (s.c.) in the right back flank with one million EG7-OVA cells. When the tumors grew to 100 mm^3^, mice were divided into 5 groups randomly (n = 7–13), and administrated in different ways: Saline, free 2DG plus Met (containing 0.4 mg 2DG, 0.2 mg Met and injection every 3 days), HAuNS-Met@MS plus NIR laser (MS ( +) NIR, containing 2 mg Met and injection for one time), MS ( +) NIR in combination with intermittent administration of 2DG ( MS ( +) NIR plus 2DG (i), HAuNS-Met@MS containing 2 mg Met was injected for one time and 2DG was injected every 3 days at dosage of 0.4 mg), and MS ( +) NIR in combination with continuous administration of 2DG ( MS ( +) NIR plus 2DG (c), HAuNS-Met@MS containing 2 mg Met was injected for one time and 2DG was injected every day at dosage of 0.4 mg). After two weeks’ vaccine, mice were sacrificed, LNs and spleen were isolated and digested to single cell suspension for immune assay.

### Anti-metastatic tumor efficacy with MS ( +) NIR plus 2DG in vivo

Mice were divided into 5 groups (n = 9) randomly, and s.c injected with one million E.G7-OVA tumor cells. After 6 to 10 days, tumors formed and the mice were administrated with above mentioned dosing strategy. After 10 days’ treatment, mice were challenged with second tumor implantation as distant tumor. The body weight and tumor volume of each mouse were monitored every 2 days for 3 weeks’ treatment. The tumor volumes were calculated according to the following formula: a*b*c*0.5 where a is the major axis, b is the minor axis and c is the height-diameter of the tumor. On day 24, all the mice were sacrificed and tumor tissues including primary tumor, distant tumor and the metastatic tumor nodules under the whole skin of mice were collected, weighed, and photographed. Immune cells were isolated from lymph node or spleen and analyzed with flow cytometry.

### Anti-B16F10 tumor efficacy with MS ( +) NIR plus 2DG in vivo

Mice were divided into 7 groups (n = 13), and s.c injected with one million B16F10 tumor cells. When the tumor sizes were about 100 mm3, mice were randomly divided into 4 groups (n = 14): (1) Saline; (2) 2DG plus Met; (3) MS ( +) NIR; (4) MS ( +) NIR plus 2DG (i); (5) MS ( +) NIR plus 2DG (c). 24 h later, the mice in the laser group were irradiated with an 808 nm laser (2 W cm-2) for 3 min. Two weeks later, half of the mice were sacrificed to evaluate the immune situation. The remaining mice continued to receive treatment and tumor changes were observed. Two weeks later, all mice were sacrificed to collect the tumors, followed by tumor weighing and photographing. The relative tumor volumes were calculated.

### Cytokine detection

The spleen tissue, LNs, intratumor, and plasma level of IFN-γ, granzyme B, IL-2, IL-4, IL-6, IL7Rα, CCR7, IL-15 and CD62L were measured with ELISA kits according to the manufactures’ instructions.

### Immunofluorescence staining and immunohistochemical assay

The fresh tumor tissues and other normal organs were fixed in 4% paraformaldehyde, embedded in paraffin, cut into 5 μm slices, and stained with different primary antibodies such as CD8, IFN-γ and granzyme B overnight at 4 °C following the manufacturer's instructions. After the addition of fluorescently labelled secondary antibodies. The slides were analyzed with a fluorescence microscope (Nikon, Japan). Immunofluorescence staining of tumor cells were studied as previous reports. H&E, Ki67 and TUNEL results of different tissues were determined with immunohistochemical assay.

### Western blotting

Tumor cells and T cells were treated with different methods, after that, cells were collected and lysed with RIPA. Equal amount of protein measured using BCA Protein Assay Kit were mixed with loading buffer and boiled at 90 °C for 10 min. The expression of CRT, HMGB-1, HSP70, p-AMPKα, mTOR, ACC1 and CPT2 were determined by western blot. The procedures of western blotting and protein extraction referred to our previous studies [16].

### Flow cytometric assay

Mice were sacrificed, and the tumors, spleens, and LNs were isolated and minced using surgical scissors. Tissues were digested with Collagenase II and then passed through a 40-mm filter. After three rounds of PBS washes, single-cell suspensions were harvested and then subjected to fluorescein-conjugated staining. For intracellular staining (such as IFN-γ andFoxp3), samples were incubated with the penetration buffer in BD Cytofix/ Cytoperm Kit followed by incubation with antibodies according to the manufacturer’s protocols. The preliminary FSC/SSC gates of the starting cell population were set based on the size of lymphocytes. All samples were subject to flow cytometry (BD Fortessa, Becton Dickinson Company, MA) and analyzed with the FlowJo software.

### Statistical analysis

All the data were displayed as representative or the results from multiple independent experiments. The data comparisons were achieved by Student's *t* test and one-way ANOVA test. P* < 0.05 was regarded as statistical significance. P** < 0.01 was considered as extreme statistical significance. All error bars are expressed as ± SD.

## Supplementary Information

**Additional file 1**: **Figure S1**. The hydrated particle size of MS measured by dynamic light scattering (A) and in vitro cytotoxicity of HAuNS-Met@MS at various concentration. **Figure S2**. The standard curve of Met through HPLC. **Figure S3**. Proliferation curve of CD8+T cell. **Figure S4**. (A) IC50 of free 2DG, Met and 2DG plus Met on B16F10 cells. (B) Cell viability of B16F10 after being incubated with free 2DG and Met at different concentration. **Figure S5**. Mitochondrial membrane potential fluorescence of B16F10 tumor cells. **Figure S6**. (A) Mitochondrial membrane potential fluorescence of EG7-OVA tumor cells. (B) Flow cytometry plot of mitochondrial membrane potential fluorescence. (C) Quantification based on (B). **Figure S7**. Representative flow cytometry plots (A) and quantification (B) of apoptosis rates of EG7-OVA cells. **Figure S8**. Flow cytometry analysis of DC maturation by analyzing CD11c+MHCI+ DCs. **Figure S9**. H&E staining of skin, lymph nodes, and tumor tissues isolated from B16F10 tumor bearing mice, the red arrows indicate areas of inflammation. **Figure S10**. Treatment and administration strategy. **Figure S11**. (A) Flow cytometry plots of DC and OVA-specific DC in lymph nodes of EG7-OVA mice, (B) Quantification of flow cytometry based on (A). **Figure S12**. Flow cytometry of lymphocytes (A), CD3+T (B) and CD3+CD8+T cells (C) in lymph nodes of EG7-OVA mice. **Figure S13**. Flow cytometry plots of OVA-specific CD8+T cells in lymph nodes (A), PBMC (B) and spleen (C) of EG7-OVA mice. **Figure S14**. Flow cytometry of CD3+T cells in tumor of EG7-OVA mice. **Figure S15**. EG7-OVA tumor model on C57 mice. **Figure S16**. B16F10 tumor model on C57 mice. **Figure S17**. H&E assays of tumor cells in the MS (+) NIR plus 2DG group. **Figure S18**. Flow cytometry plots of CD8+ T cells in LNs, spleen and PBMCs after different treatments. **Figure S19**. Immunofluorescence images of Treg cells and functional CD8+T cells infiltrated tumor sections after different treatments (A); (B) Quantitation of Treg cells based on flow cytometry; (C) Quantitation of Treg cells based on immunofluorescence images.

## Data Availability

The data and materials of the study can be obtained from the corresponding author upon request.
