# Supplementary material for: Regulating immune memory and reversing tumor thermotolerance through a step-by-step starving-photothermal therapy
Source: J Nanobiotechnology. 2021 Sep 30;19:297. doi: 10.1186/s12951-021-01011-2 (PMC8482573; doi:10.1186/s12951-021-01011-2)
Supplement: Supplementary file 1 — Additional file 1: Figure S1. The hydrated particle size of MS measured by dynamic light scattering (A) and in vitro cytotoxicity of HAuNS-Met@MS at various concentration. Figure S2. The standard curve of Met through HPLC. Figure S3. Proliferation curve of CD8+T cell. Figure S4. (A) IC50 of free 2DG, Met and 2DG plus Met on B16F10 cells. (B) Cell viability of B16F10 after being incubated with free 2DG and Met at different concentration. Figure S5. Mitochondrial membrane potential fluorescence of B16F10 tumor cells. Figure S6. (A) Mitochondrial membrane potential fluorescence of EG7-OVA tumor cells. (B) Flow cytometry plot of mitochondrial membrane potential fluorescence. (C) Quantification based on (B). Figure S7. Representative flow cytometry plots (A) and quantification (B) of apoptosis rates of EG7-OVA cells. Figure S8. Flow cytometry analysis of DC maturation by analyzing CD11c+MHCI+ DCs. Figure S9. H&E staining of skin, lymph nodes, and tumor tissues isolated from B16F10 tumor bearing mice, the red arrows indicate areas of inflammation. Figure S10. Treatment and administration strategy. Figure S11. (A) Flow cytometry plots of DC and OVA-specific DC in lymph nodes of EG7-OVA mice, (B) Quantification of flow cytometry based on (A). Figure S12. Flow cytometry of lymphocytes (A), CD3+T (B) and CD3+CD8+T cells (C) in lymph nodes of EG7-OVA mice. Figure S13. Flow cytometry plots of OVA-specific CD8+T cells in lymph nodes (A), PBMC (B) and spleen (C) of EG7-OVA mice. Figure S14. Flow cytometry of CD3+T cells in tumor of EG7-OVA mice. Figure S15. EG7-OVA tumor model on C57 mice. Figure S16. B16F10 tumor model on C57 mice. Figure S17. H&E assays of tumor cells in the MS (+) NIR plus 2DG group. Figure S18. Flow cytometry plots of CD8+ T cells in LNs, spleen and PBMCs after different treatments. Figure S19. Immunofluorescence images of Treg cells and functional CD8+T cells infiltrated tumor sections after different treatments (A); (B) Quantitation of Treg cells based on f [file 12951_2021_1011_MOESM1_ESM.docx]

**Figure S1.** The hydrated particle size of MS measured by dynamic light scattering (A) and in vitro cytotoxicity of HAuNS-Met@MS at various concentration. **Figure S2.** The standard curve of Met through HPLC.**Figure S3.** Proliferation curve of CD8+T cell.**Figure S4.** (A) IC50 of free 2DG, Met and 2DG plus Met on B16F10 cells. (B) Cell viability of B16F10 after being incubated with free 2DG and Met at different concentration.**Figure S5.** Mitochondrial membrane potential fluorescence of B16F10 tumor cells.**Figure S6.** （A）Mitochondrial membrane potential fluorescence of EG7-OVA tumor cells. (B) Flow cytometry plot of mitochondrial membrane potential fluorescence. (C) Quantification based on (B).**Figure S7.** Representative flow cytometry plots (A) and quantification (B) of apoptosis rates of EG7-OVA cells.**Figure S8.** Flow cytometry analysis of DC maturation by analyzing CD11c+MHCI+ DCs.**Figure S9.** H&E staining of skin, lymph nodes, and tumor tissues isolated from B16F10 tumor bearing mice, the red arrows indicate areas of inflammation. **Figure S10.** Treatment and administration strategy.**Figure S11**. (A) Flow cytometry plots of DC and OVA-specific DC in lymph nodes of EG7-OVA mice, (B) Quantification of flow cytometry based on (A).**Figure S12**.Fow cytometry of lymphocytes (A), CD3+T (B) and CD3+CD8+T cells (C) in lymph nodes of EG7-OVA mice. **Figure S13.** Flow cytometry plots of OVA-specific CD8+T cells in lymph nodes (A), PBMC (B) and spleen (C) of EG7-OVA mice. **Figure S14.** Flow cytometry of CD3+T cells in tumor of EG7-OVA mice. **Figure S15.** EG7-OVA tumor model on C57 mice.**Figure S16.** B16F10 tumor model on C57 mice.**Figure S17.** H&E assays of tumor cells in the MS (+) NIR plus 2DG group.**Figure S18.** Flow cytometry plots of CD8+ T cells in LNs, spleen and PBMCs after different treatments.**Figure S19.** Immunofluorescence images of Treg cells and functional CD8+T cells infiltrated tumor sections after different treatments (A); (B) Quantitation of Treg cells based on flow cytometry; (C) Quantitation of Treg cells based on immunofluorescence images.
